# Global climate change below 2 °C avoids large end century increases in burned area in Canada

**DOI:** 10.1038/s41612-024-00781-4

**Published:** 2024-10-01

**Authors:** Salvatore R. Curasi, Joe R. Melton, Vivek K. Arora, Elyn R. Humphreys, Cynthia H. Whaley

**Affiliations:** 1 Canadian Centre for Climate Modelling and Analysis, Environment and Climate Change Canada, Victoria, BC Canada; 2 Climate Research Division, Environment and Climate Change Canada, Victoria, BC Canada; 3 Department of Geography & Environmental Studies, Carleton University, Ottawa, ON Canada

**Keywords:** Climate sciences, Environmental sciences

## Abstract

Wildfire impacts the global carbon cycle, property, harvestable timber, and public health. Canada saw a record fire season in 2023 with 14.9 Mha burned—over seven times the 1986–2022 average of 2.1 Mha. Here we utilize a new process-based wildfire module that explicitly represents fire weather, fuel type and availability, ignition sources, fire suppression, and vegetation’s climate response to project the future of wildfire in Canada. Under rapid climate change (shared socioeconomic pathway [SSP] 370 & 585) simulated annual burned area in the 2090 s reaches 10.2 ± 2.1 to 11.7 ± 2.4 Mha, approaching the 2023 fire season total. However, climate change below a 2 °C global target (SSP126), keeps the 2090 s area burned near modern (2004–2014) norms. The simulated area burned and carbon emissions are most sensitive to climate drivers and lightning but future lightning activity is a key uncertainty.

## Introduction

Rapidly rising temperatures and changing precipitation regimes are resulting in regional increases of boreal wildfire^1–4^ in Canada and elsewhere. In Canada, the size and frequency of large wildfires (>200 ha) is increasing as is the length of the fire season^2,3^. The 2023 fire season in Canada burned an unprecedented 14.9 Mha, or roughly seven times the 1986–2022 average^5^, which largely drove a 24% increase in global tree cover loss^6^. Several other circumpolar countries have reported record fire seasons. In 2021, 14.3 Mha of forest burned in Russia while in 2018, 0.02 Mha burned in Sweden^7–9^. This increase in wildfire is important as it impacts the landscape’s ecosystems, human population, and the carbon (C) cycle with potentially important feedbacks to the climate system. Wildfire events can lead to costly suppression efforts, property damage, and loss of life. Fire suppression efforts in Canada cost between $702 million and $1.23 billion annually (2007–2017 in 2009 CAD)^10,11^ while, between 1970 and 2009, individual large fire events incurred total insurance claims between $221 million and $3.36 billion (in 2009 CAD)^12^. Volatile gasses emitted by wildfires can also have both acute and long-term detrimental human health impacts across the country and over borders^5,13,14^. Finally, wildfire transfers carbon from vegetation, soil, and litter to the atmosphere thus impacting the annual net carbon balance of the landscape^15–17^. For Canada’s managed forests, at decadal timescales, fire impacts forest age classes, and structure and thus influences their merchantability^18,19^. Projections of the broad patterns of future Canadian wildfire under climate change are key to understanding and mitigating its potential impacts.

Representing wildfires, and in particular boreal fires, in land surface models (LSMs) is challenging due to our limited understanding of the processes involved^20–22^. The spatial distribution and trends in wildfires in Canada and other northern regions result from a complex interplay between climate, fuel type, fuel availability, ignition sources (e.g., lightning vs. human ignitions), and fire suppression^1–4,12,23–25^. Nevertheless, global area burned can be skilfully represented by LSMs that model wildfire based on different combinations of atmospheric drivers, simulated soil moisture, lightning ignition, anthropogenic drivers, and fuel availability ^26^. This may be because ~90% of global burned area and wildfire emissions occur in the tropics where fire is driven by periods of low soil moisture and extensive lightning^21,26^. In contrast, LSMs generally underestimate burned area in high-latitude boreal regions like Canada where other drivers including relative humidity are important^21,22,27,28^. Fire risk indices like the Canadian forest fire weather index system (FWI) have been successfully used in Canada and other parts of the world for decades to provide a proxy for fire activity based on meteorological drivers. They have also been used to evaluate the frequency of extreme fire events^29–31^. However, FWI is only a proxy for wildfire risk/severity and does not directly yield burned area^32^. Operational fire models accurately predict the progression of individual fire events at high resolution to support decision-making and fire suppression efforts^28,33^. However, because they rely on real-time observation, they are not well suited to making long-term projections at continental scales. Other classes of models, including data-driven models, can predict burned areas in Canada^19,32,34,35^. However, these models are unable to estimate wildfire emissions or dynamically represent the complex feedback between wildfire, vegetation, and climate change^4,19,24,25,32,34,35^. There are, to our knowledge, no high-resolution, spatially explicit, process-based projections of future area burned for Canada.

Here we investigate the response of wildfires to climate change in Canada. We implement and evaluate a new wildfire module in an LSM tailored to Canada, the Canadian Land Surface Scheme Including Biogeochemical Cycles (CLASSIC)^22^, which is the terrestrial component of the Canadian Earth System Model (CanESM)^36^. The LSM’s default fire module has been evaluated at the global scale and successfully reproduces the broad geographical distribution of area burned driven primarily by climate and historical trends driven primarily by changes in population density^20^. However, this default fire module greatly underestimates the historical burned area in Canada. To better represent the short-term climatic controls on fire ignition and spread in Canada, we integrate aspects of FWI^37–40^ and the Canadian Forest Fire Behavior Prediction (FBP) System^41^ (see “Methods”) in the framework of the CLASSIC LSM. The updated LSM explicitly represents feedbacks between climate, vegetation biomass, anthropogenic ignition/suppression, and lightning ignitions. In this study, we assess the fire module’s performance when driven by climate reanalysis and observed lightning strike frequencies through the 1985–2015 period. We utilize three remotely sensed burned area reference datasets and five fire CO_2_ emissions estimates to demonstrate that the model is capable of simulating the temporal trajectories of these variables over the historical period and quantifying any associated biases^42^ (Table 1). To understand the response of Canadian wildfire to climate change, we project future area burned using bias-corrected Inter-Sectoral Impact Model Intercomparison Project (ISIMIP) climate forcings from six earth system models^43^ and projected lightning strike frequencies from two models^44,45^ under three shared socioeconomic pathways (SSP126, SSP370, and SSP585). Finally, to enhance our understanding of future wildfire drivers, we analyze the module’s sensitivity to different forcings through factorial analysis.

**Table 1:** Summary of the satellite-based burned area (BA) and fire CO_2_ emissions (fire CO_2_) datasets used to evaluate the simulated results

Dataset	Variables	Timestep	Period evaluated	Reference	Method
GFED4.1s	BA; fire- CO_2_	Monthly	2001–2015; 2003–2015	Giglio et al.^111^	Remotely sensed (MODIS) burned area mapping with small fire estimation; bottom-up emissions modeling utilizing CASA.
MODIS	BA	Monthly	2001–2015	Chuvieco et al.^112^	Remotely sensed (MODIS) burned area mapping.
NTEMS	BA	Annual	1985–2017	Hermosilla et al.^113,114^	Remotely sensed (LANDSAT) burned area mapping.
FINN2.5	Fire CO_2_	Monthly	2003–2015	Wiedinmyer et al.^55^	Bottom-up emissions modeling utilizing MODIS active fire data.
FEER1.0-G1.2	Fire CO_2_	Monthly	2003–2015	Ichoku and Ellison^57^	Top-down fire radiative power emissions estimation.
QFED2.4r1	Fire CO_2_	Monthly	2003–2015	Koster et al.^58^	Top-down fire radiative power emissions estimation.
CT2019	Fire CO_2_	Monthly	2003–2015	van der Werf et al.^54^	Bottom-up emissions modeling utilizing CASA.

The datasets include the Global Fire Emissions Database version 4.1 with small fires (GFED4.1 s), European Space Agency Climate Change Initiative FireCCI50 MODIS derived burned area (MODIS), the National Terrestrial Ecosystem Monitoring System for Canada (NTEMS), the Global Fire Emissions Database version 4.1 with small fires (GFED4.1 s), the Fire Inventory from NCAR version 2.5 (FINN2.5), Fire Energetics and Emissions Research version 1.0-G1.2 (FEER1.0-G1.2), the Quick Fire Emissions Dataset version 2.4 revision 1 (QFED2.4r1), and Carbon Tracker 2019 (CT2019).

## Results

### Fuel moisture and lightning are key to modeling Canadian wildfire

**Fig. 1:**
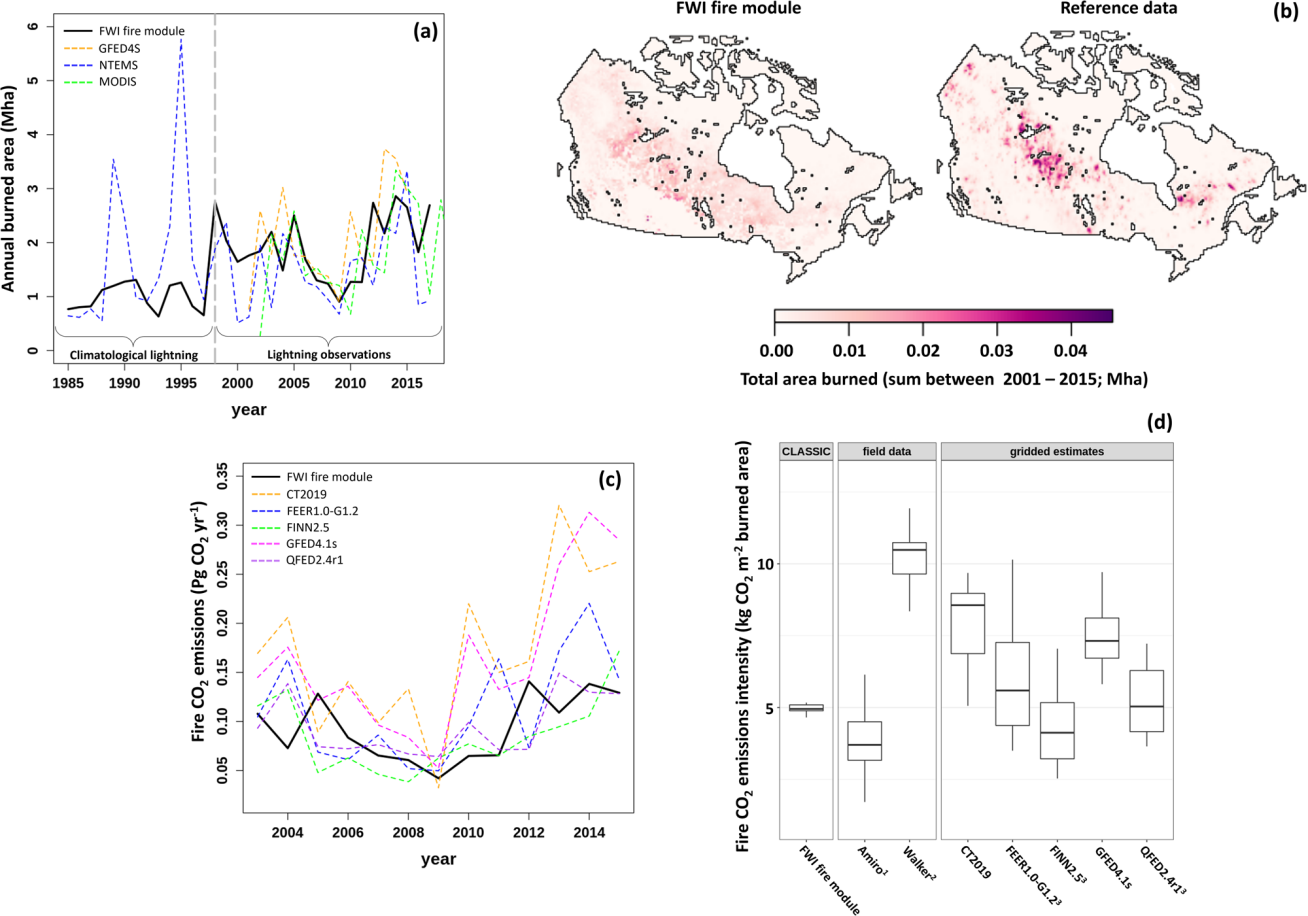
Comparisons between results from the fire weather index (FWI) based fire module of the CLASSIC model and reference datasets. **a** Annual time series of burned area for the FWI-based fire module driven by bias-corrected reanalysis compared to three satellite-based reference datasets. **b** The spatial distribution of total burned area from the FWI-based fire module compared to the mean of total burned area (2001–2015) from three satellite-based reference datasets. **c** The annual time series of fire CO_2_ emission from the FWI-based fire module compared to five bottom-up and top-down fire CO_2_ emissions estimates. **d** Fire emissions intensities for those same five emissions estimates, as well as published field datasets. The reference datasets come from the Global Fire Emissions Database version 4.1 with small fires (GFED4.1 s), European Space Agency Climate Change Initiative FireCCI50 MODIS derived burned area (MODIS), the National Terrestrial Ecosystem Monitoring System for Canada (NTEMS), the Fire Inventory from NCAR version 2.5 (FINN2.5), Fire Energetics and Emissions Research version 1.0-G1.2 (FEER1.0-G1.2), the Quick Fire Emissions Dataset version 2.4 revision 1 (QFED2.4r1), and Carbon Tracker 2019 (CT2019). In panel “d” the line, bar and whiskers represent the 50th percentile, 25th–75th percentile, maximum, and minimum respectively. ^1^See Table 2 in ref. ^52^ for regional means and weights. ^2^See ref. ^4^. Ecoregion means excluding Alaskan sites. ^3^For top-down (i.e., FEER, QFED) and fire anomaly-based data (FINN2.5) estimates, fire CO_2_ emissions are normalized by all three annual burned area estimates in (**a**) with the resultant uncertainty propagated into the box plot.

Our FWI-based module realistically represents the influence of fuel moisture content, atmospheric humidity, and ignition on wildfire. When driven by biased corrected reanalysis data and observed lightning strike frequencies, the model captures the mean annual state and interannual variability of burned area reasonably well as compared to three satellite-based reference datasets. Between 2001 and 2015 mean annual modeled burned area is 1.86 Mha compared to 1.83 ± 0.67 Mha (Mean ± Std. Err.) for the reference datasets (Fig. 1a). The model reasonably captures the reference dataset patterns of interannual variation (Pearson correlation [*r*] = 0.48; Fig. 1a) as well as the spatial patterns (spearman correlation [*r*_s_] = 0.47; Fig. 1b).

The detailed model output must be interpreted in light of the scale at which the model drivers operate. In nature, fuel moisture and lightning ignitions are key determinants of short-term variability in the frequency and size of burned area^24,46,47^, whereas fuel availability and drought influence long-term trends in burned area^4,46,48^. In the model, when lightning observations are unavailable and we use climatological mean lightning (1985–1998; Fig. 1a), there is less interannual variability in simulated burned area.

The module represents 1985–1998 mean modeled burned area (1.11 Mha) similar to the National Terrestrial Ecosystem Monitoring System for Canada (NTEMS) reference (1.74 Mha), but captures less interannual variability (*r* = 0.28). In all of our simulations, the modeled burned area is more spatially homogeneous than the reference dataset in which 2001–2015 total burned area is concentrated in central Canadian boreal forests (Fig. 1b). This is likely in part because of the model’s Canada-specific tree plant functional types (PFTs), which require tradeoffs between realism and parsimony ^22^. The model PFTs represent the presence of highly flammable spruce genera (*Picea* spp.) that have low limbs allowing fire to propagate into the canopy, but not, for example, more subtle differences in soil organic layer thickness and dryness between black spruce (*Picea mariana*) and jack pine (*Pinus banksiana*) stands^4,22^. In addition, natural hazards are inherently stochastic. In this case, stochasticity may influence the location and timing of large fires that represent the majority of the burned area during the reference period^49,50^ and is not reproducible in a quasi-mechanistic LSM. Finally, some portion of this homogeneity may be a product of the climate drivers’ source resolution of 0.5 degrees which is coarser than the 0.22-degree model resolution.

### Wide range of CO_2_ emissions estimates for historical Canadian fires

Canadian wildfire CO_2_ emissions estimates vary widely among methods and sources^51^. Between 2003 and 2015 our modeled mean fire CO_2_ emissions (0.093 Pg CO_2_ yr^−1^) fell within the lower end of the range provided by five other spatially explicit estimates (0.085–0.172 Pg CO_2_ yr^−1^; Fig. 1c). Modeled emissions have a similar temporal trend and interannual variation when compared to the five other spatially explicit estimates (*r* = 0.52; Fig. 1c) and are tightly correlated with modeled burned area (*r* = 0.99). When normalized by burned area, our fire emission intensity (5.0 kg CO_2_ m^−2^ burned area) falls towards the center of the range of several gridded and field-based estimates (1.7–14.5 kg CO_2_ m^−2^ burned area; Fig. 1d). Our modeled fire emission intensity falls just above that of Amiro et al.^52^, derived from repeat observations at Canadian experimental fires^41^. These comparisons and correlation analysis show that the LSM can reliably simulate fire CO_2_ emissions.

The wide range of reference fire CO_2_ emissions estimates highlights uncertainties in our understanding of this process^51^. The differences (Fig. 1c) are partly due to differences in the types of fires explicitly considered in the various datasets. For example, some datasets derived using bottom-up approaches, including GFED4.1 s (Global Fire Emissions Database version 4.1 with small fires) and CT2019 (Carbon Tracker 2019), consider small fires (<500 m)^53,54^ as well as other small emissions sources like agricultural (~1.7% of emissions) and post-timber harvest burns^51,53–56^. The majority of the differences in fire CO_2_ emissions stem from the representation of emissions from belowground (soil) sources that have an outsized role in Canada as compared to lower latitudes^53,54^. Both GFED4.1 s and CT2019 have high emissions intensity and build upon the same core model (Carnegie–Ames–Stanford approach)^51^, which likely is a cause of the large proportion of fire CO_2_ emissions they derive from belowground sources (~60%) and peatland land cover (~7.3%) in boreal North America^51,53,54,56^. QFED2.4r1 (Quick Fire Emissions Dataset version 2.4 revision 1) and FEER1.0-G1.2 (Fire Energetics and Emissions Research version 1.0-G1.2) are derived from top-down approaches using smoke aerosols and thus should implicitly consider fire CO_2_ emissions from soil^51,57,58^. These datasets however have lower CO_2_ emissions and are closer to our model results than the bottom-up estimates. Finally, field-based observations that explicitly consider soil fire CO_2_ emissions span a range of intensities, similar to gridded estimates (Fig. 1d)^4,52^.

### Future fire diverges under different shared socioeconomic pathways

The LSM projects different fire futures under the different SSPs. The SSPs represent a range of future climates dependent upon storylines that make assumptions about how future energy needs are met (see Table S1 and Fig. S1). The SSP370 “Regional Rivalry” and SSP585 “Fossil-fueled Development” scenarios represent futures with energy-intensive growth and potent challenges to climate change mitigation^59^. The SSP126 “Sustainability” scenario assumes sustainable growth and net-zero/negative CO_2_ emissions around 2075 and is compatible with a global 2 °C temperature target (Fig. S1)^59^. The climate drivers used in our simulations are based on bias-corrected climate from six ESMs. This addresses the spread across the six climate models considered here, yielding an ensemble of projections well suited to assessing future trends in mean burned area and the uncertainty of the mean. However, our results are based on only one ensemble member from each climate model and do not represent the entire range of internal climate variability^5^ that climate models may exhibit. This requires the use of a large ensemble with typically more than 50 ensemble members for each climate model. Moreover, our deterministic model represents the state of a natural hazard that is inherently stochastic^50^. In our modeling framework, the only source of stochasticity introduced into the simulations is inherited from the driving climate. As a result, our results cannot be used to quantitatively assess the frequency of future extreme wildfire events.

**Fig. 2:**
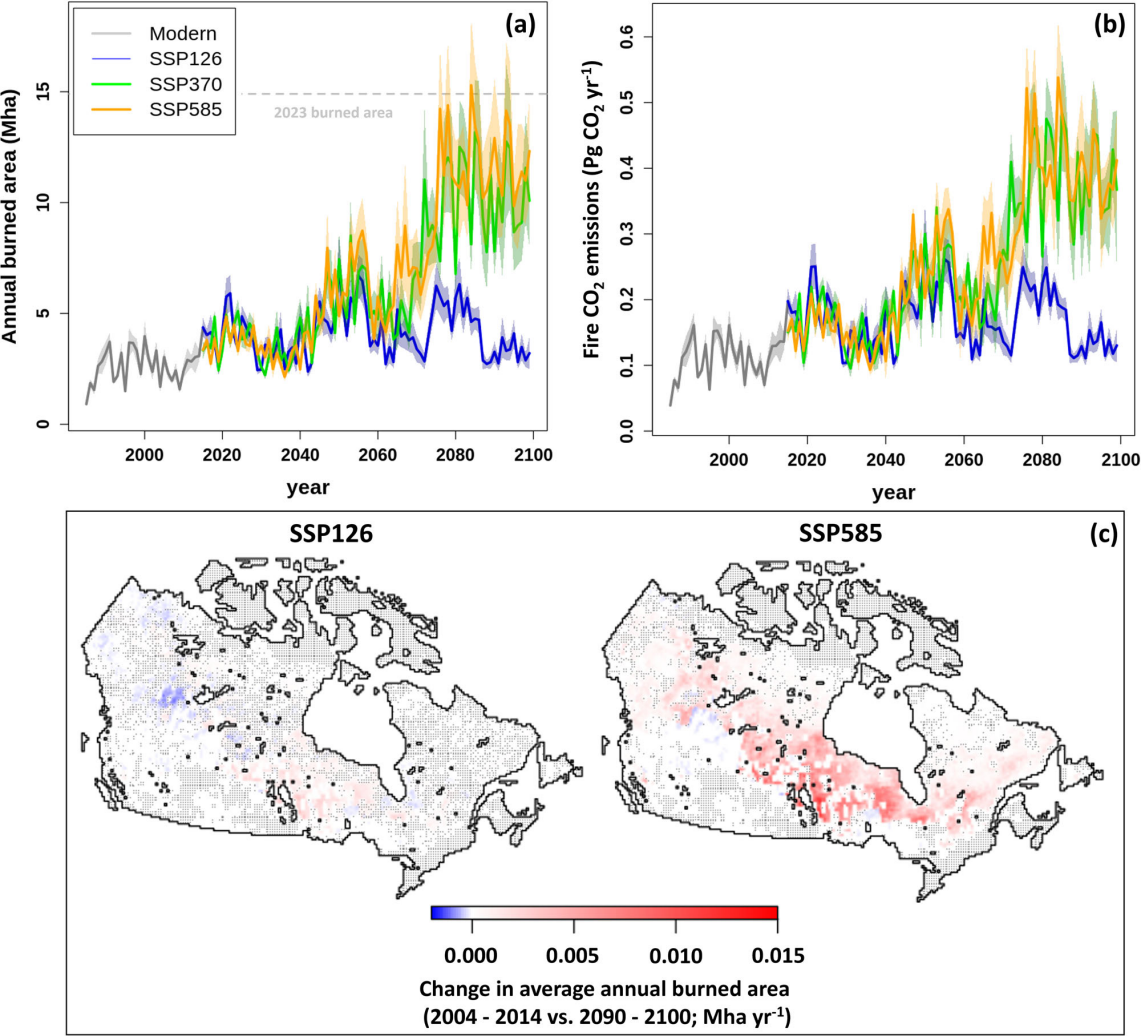
Temporal and spatial patterns of projected burned area and fire CO_2_ emissions under three shared socioeconomic pathways (SSPs). **a** Annual time series of burned area for the modern period and three SSPs with a dashed gray line indicating the burned area of the 2023 fire season. **b** Annual time series of fire CO_2_ emissions for the modern period and three SSPs. **c** Maps of the change in average annual burned area between the modern reference period (2004–2014) and the end of the century (2090–2100) for the SSP126 and SSP585 scenarios. Stippling denotes no change based on *t* tests across the ensemble members. The shaded regions denote one standard error.

We project increases in mean burned area for all three SSPs rising from 2.5 ± 0.3 Mha yr^−1^ in the modern period (2004–2014) to 4.5 ± 0.7 Mha mid-century (2040–2050; 73–105% higher than the modern period; *P* < 0.05 under all SSPs; Fig. 2a). By the end of the century (2090–2100), the projected burned area diverges for the three SSPs. Burned area plateaus at 282–414% higher than the modern period under SSP370 (10.2 ± 2.1 Mha; *P* < 0.01) and SSP585 (11.7 ± 2.4 Mha; *P* < 0.01). For context, the record 2023 fire season burned 14.9 Mha^5^. Most of the increased burned area occurs throughout Canada’s boreal region (Fig. 2c). Under SSP126, burned area at the end of the century is not significantly different from the burned area simulated for 2004–2014 (3.3 ± 0.5 Mha; *P* > 0.05) with little significant change in distribution across Canada (Fig. 2c). The projection uncertainty (i.e., the shading in Fig. 2b) is attributable to the six ESM climates (37%), the two lightning drivers (22%), and climate and lightning acting concurrently (41%).

The LSM projects increase in fire CO_2_ emissions (64–96%; *P* < 0.05) under all SSPs between the modern period (0.109 ± 0.014 Pg CO_2_ yr^−1^) and mid-century (0.190 ± 0.026 Pg CO_2_ yr^−1^; Fig. 2b). Fire CO_2_ emissions increase further by the end of the century reaching a plateau at 226–276% higher under SSP370 (0.378 ± 0.069 Pg CO_2_ yr^−1^; *P* < 0.01) and SSP585 (0.390 ± 0.063 Pg CO_2_ yr^−1^; *P* < 0.001). Under SSP126, fire CO_2_ emissions are similar to modeled modern values (0.131 ± 0.016 Pg CO_2_ yr^−1^; *P* > 0.05).

Our results explicitly consider fire weather, lightning strike frequencies, anthropogenic ignition/suppression, vegetation carbon stocks, and vegetation’s response to climate and CO_2_ fertilization^19,32,34,35^. Since 1959 when reliable records became available, area burned has increased in Canada and is attributed to increased lightning fire ignitions^1,2,60^. Large fires (>200 ha), represent the majority of burned area and are increasing in size and frequency throughout Canada^1–3^. Based on our projections, burned area, and fire CO_2_ emissions will continue increasing two- to fourfold by the end of the century under rapid climate change (SSP370/585) primarily in the forested Canadian Shield, interior plains, and western cordillera. However, under a tempered scenario (SSP126), these increases can be avoided. Under SSP126, fire patterns in Canada remain relatively stable as atmospheric CO_2_ concentrations, mean annual temperature, and precipitation plateau near 2050, and lightning strike frequency remain stable.

### Climate and lightning determine future fire trajectories

**Fig. 3:**
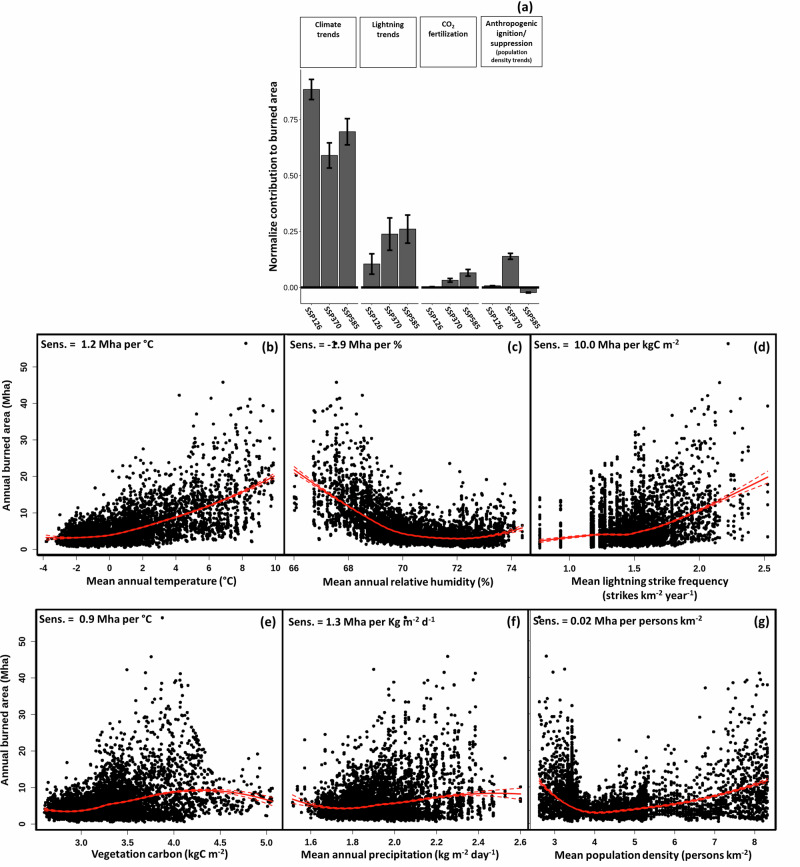
The sensitivity of projected Canada-wide burned area to primary drivers from all future and factorial simulations. **a** Contribution of climate trends, lightning trends, CO_2_ fertilization, and anthropogenic ignition/suppression (population density) to the burned area are normalized from 0 (0% of the trend) to 1 (100% of the trend). The dependence between annual burned area and **b** Canada-wide mean annual temperature. **c** Canada-wide mean annual relative humidity. **d** Canada-wide mean annual lightning strike frequency. **e** Canada-wide mean annual vegetation carbon. **f** Canada-wide mean annual precipitation. **g** Canada-wide mean annual population density. The plots visualize all 90 simulations from 2014 to 2100 (*n* = 7740 points visualized) and include a LOESS regression line in red with 95% confidence intervals. Error bars denote one standard error.

To gain a more detailed understanding of future wildfire drivers, we analyze the sensitivity of burned area and fire CO_2_ emissions to climate, lightning strike frequency, human population density, CO_2_ fertilization, and vegetation biomass (Fig. 3). Factorial analysis of the 2090 s modeled burned area over the last decade of the century indicates that climate is the largest contributor to burned area trends across the simulations (72 ± 4% under all SSPs). The effects of climate on fire are primarily due to changes in temperature and humidity whose mean annual Canada-wide values are strongly correlated (Table S2; Figs. 3, S1b, e, and S2). The sensitivity of burned area to Canada-wide mean annual temperature based upon all future and factorial simulations (90 simulations from 2014 to 2100; *n* = 7740 points visualized; Table S1) is 1.2 Mha °C^−1^. The sensitivity to relative humidity is 1.9 Mha %^−1^ (Fig. 3b, c). FWI captures the strong non-linear influence of relative humidity on the drying rates of upper soil and duff fuel layers^61^. Moreover, although FWI does not explicitly include vapor pressure deficit (VPD), our model exhibits an emergent (not specified apriori) sensitivity to VPD mirroring in situ results (Fig. S2)^62,63^.

The next most important contributor to burned area trends is lightning (21 ± 4%) with a sensitivity of 10.0 Mha strikes^−1^ km^−2^ year^−1^ (Fig. 3d). In nature, and in our modeling framework, these two drivers along with relative humidity directly influence fire’s ignition probability and, in the case of temperature, its spread rate^1,2,38,41,45,47^. The contribution and sensitivity for other forcings like anthropogenic ignition/suppression (population density) is much lower at 4 ± 1% or 0.02 Mha strike^−1^ km^−2^ year^−1^ (Fig. 3g). Our modeling framework captures changes in anthropogenic ignition/suppression related to human land use through population density, and assumes that future fire management practices remain unchanged (see: “Methods”, Eq. (S1)).

The trends in fire CO_2_ emissions are similar, albeit with a stronger contribution from vegetation biomass (due to CO_2_ fertilization) and a weaker lightning contribution (Figs. S2b and S3a). The sensitivities for fire CO_2_ emissions also exhibit similar patterns (Fig. S3b–g). However, vegetation biomass has a relatively larger impact on fire CO_2_ emissions as vegetation, soil, and litter are the source pools for emissions^20,52,64–66^.

## Discussion

Climate change will strongly influence fire in Canada. How lightning patterns will change is particularly important to fire patterns but also highly uncertain^1,45,46,60^. In the future, model drivers derived from large ensemble climate projections that represent the full range of internal climate variability will allow for a greater understanding of future boreal wildfire extremes^50,67^. High-resolution spatially explicit lightning time series, like those used herein, are key to improving the projections of Canadian and boreal wildfire^44,45,68^. More extensive lightning observation networks and detailed representation of lightning in ESMs may increase the certainty of wildfire projections and the representation of feedbacks between climate, lightning, and fire^44,47^.

The LSM’s FWI fire model used here is of intermediate complexity. Incorporating additional processes could improve our LSM’s representation of fire emissions and burned area. These additional processes could include consideration of fire severity (e.g., smoldering versus crown fire), depth of fire burn, vegetation fuel quality, disturbance-mediated subgrid-scale heterogeneity, shifts in vegetation cover, nutrient cycling, peatland hydrology, peatland soils, overwintering fire, landscape fragmentation, land use change, management practice that impact ignition sources, and fire suppression policy^4,12,20,48,69–78^. Nevertheless, by representing the influence of fuel moisture content, atmospheric humidity, and ignition on wildfire our FWI-based module realistically represents wildfire in Canada. CLASSIC is the land surface component of CanESM (1–2.8° resolution global domain), and CanRCM (0.11–0.22° resolution arctic domain)^65,79^ meaning this module can be applied to other circumboreal regions (i.e., Scandinavia and Eurasia) to quantify the impact of climate on boreal wildfire in the future.

Under rapid climate change (SSP585) large ensemble climate models project longer fire seasons, and more extreme fire weather (FWI)^80,81^. Our results demonstrate that Canada-wide burned area is highly correlated with FWI. It follows that more extreme fire weather conditions will lead to more frequent, and more extreme Canada-wide burned area in the future. Carbon emissions and tree cover loss due to wildfire will have feedbacks to global carbon cycling and climate change. Wildfire will impact forest age classes, structure, and merchantability. Under rapid climate change, wildfire emissions would dominate Canada’s anthropogenic emissions by the end of the century. Under the pessimistic assumption that Canada’s anthropogenic emissions remain stable at the 2022 level of 708 Tg CO_2_ equivalent year^−1^, our projected fire emissions of 378–390 Tg CO_2_ yr^−1^ in the 2090 s for the SSP370 and SSP585 scenarios would be equal to about 54% of Canada’s anthropogenic GHG emissions (1 Mt CO_2_ eq = 1 Tg CO_2_ eq)^82^. Our emissions estimates may underestimate this percentage as they exclude greenhouse gases other than CO_2_ and because other emission intensity estimates, which cover a wide range, are up to 1.6 times higher than our estimate (see “Wide range of CO_2_ emissions estimates for historical Canadian fires”). In 2023, over 200 Canadian communities (~232,000 people) were evacuated due to wildfire, and wildfire air quality impacts spread as far as the United States and Western Europe^5^. These and other costly negative implications would be expected to worsen under rapid climate change. These results are a powerful tool for informing management interventions under these rapid climate change scenarios. However, by meeting a global 2 °C temperature target above the pre-industrial level (i.e., the SSP126 “Sustainability” scenario), wildfire and its impacts are not expected to exceed current norms in Canada by the end of the century.

## Methods

### CLASSIC FWI fire module

CLASSIC is an open-source community model. It couples the Canadian Land Surface Scheme (CLASS; physics)^83–86^ and the Canadian Terrestrial Ecosystem Model (CTEM; biogeochemistry)^65,87^. The site-level performance of CLASSIC v1.0 was evaluated by Melton et al.^88^, the global performance was evaluated by Seiler et al.^79^, and model updates and improvements since v1.0 are detailed in refs. ^89–91^, and ^22,77^. We carry out simulations of the pan-Canadian domain at 0.22° spatial resolution using 14 biogeochemical plant functional types (PFTs) suitable for Canada^22^ and reflective of the boreal vegetation of other circumboreal regions.

The FWI fire module builds upon the pre-existing default fire module within CLASSIC^20,65^ by integrating aspects of the Canadian forest fire weather index system (FWI)^37–40^ and the Canadian forest fire behavior prediction (FBP) system^41^. The sensitivity of the FWI system to meteorological drivers and its representation of moisture loss in forest fuels is explored in detail in Laswon and Armitage^61^. The FWI fire module runs at the daily timestep along with the rest of CLASSIC biogeochemistry (excluding photosynthesis and leaf maintenance respiration that operate on a timestep of 30 min along with CLASSIC physics) and takes into account daily maximum air temperature, relative humidity, wind speed, and total rainfall. The FWI fire module then calculates the fine fuel moisture code (FFMC), initial spread index (ISI), and buildup index (BUI) to represent the moisture content of the various soil layers and the corresponding impacts on fire spread. The FWI calculations are implemented in CLASSIC based upon the most recent code for calculating FWI available (Information Report NOR-X-424 version) and verified against data samples provided in the original publications, and fire weather normal maps^40,92^. The module dramatically improves upon CLASSIC’s default fire module, which yields unrealistically low burned area for high-latitude regions when compared against remotely sensed benchmark data^22^.

The fire module of CLASSIC represents both natural and anthropogenic fire through a process-based scheme conditioned upon fuel availability, fuel combustibility, and the presence of an ignition source. The probability of fire occurrence (*P*_f_) for a representative area (a_rep_) of 800 km^2^ is the product of the probabilities of fire conditional on aboveground biomass (*P*_b_), field moisture content (*P*_m_), and ignition (*P*_i_ ; Eq. (1)).

1 $${P}_{f}={P}_{b}{P}_{i}{P}_{m}$$

*P*_b_ and *P*_i_ remain unchanged compared to the CLASSIC default fire module^20,65^. P_b_ varies linearly from 0 to 1 as a function of aboveground biomass between upper and lower thresholds of 0.2 and 1.0 kg C m^−2^. *P*_i_ is calculated as the cumulative contribution of both natural lightning-driven and anthropogenic ignition sources (Eq. (S1)). The probability of fire conditional on natural ignitions varies from 0 to 1 depending upon the number of cloud-to-ground lightning strikes in a particular location between upper and lower thresholds of 0.25 and 10 strikes km^−2^ year^−1^. The probability of fire conditional on human ignitions is dependent upon population density and approaches one at a population density threshold of 300 people km^−2^.

*P*_m_ incorporates the FWI scheme. *P*_m_ is a logistic function of the FFMC. The FFMC is an index between zero and one hundred that represents the moisture content of needle litter on the soil surface based upon a kinetic drying model with a time lag of 1 day. Decreasing FFMC values represent increasingly dry surface litter. The logistic *P*_m_ function is adapted from Blackmarr^93^ and asymptotes at 0 above an approximate upper threshold of 78.5, increases from 0 to 1 as FFMC decreases, and asymptotes at 1 below an approximate lower threshold of 66.7 (Eq. (2); Fig. S4).

2 $${P}_{m}=\frac{{e}^{-25.2+0.34\,{FFMC}}}{1+{e}^{-25.2+0.34\,{FFMC}}}$$

The area burned by a fire in one day (a_1,α_) is calculated assuming that fire spreads in an elliptical shape perpendicular to the wind direction. The length-to-breadth ratio of the ellipse, which is a function of wind speed, remains unchanged compared to the CLASSIC’s default fire module^20,65^.

The fire spread rate in the downwind direction (v_d_), which determines the length of the ellipse, is based on the FWI scheme. v_d_ is a vegetation type-specific function of the ISI and BUI (Eq. (3)). The equations that determine fire spread rates are derived from the Canadian FBP system^41^. They use ISI to represent the increase in fire spread rate due to higher wind speeds and dry duff fuel and BUI to represent the potential for intensification of fire in dry upper soil layers and vegetation. The ISI and BUI both range from zero to infinity. Therefore the fire spread rate is a function of the maximum spread rate for a given vegetation type (v_d,max_; km h^−1^) and two scalars which vary from zero to one as a function of ISI and BUI. The scalars follow from equations 26 and 54 in Canada Fire Danger Group^41^ normalized by their respective limits as ISI and BUI approach infinity. For grasses, the spread rate in the downwind direction is a function of ISI alone. However, it also accounts for the higher flammability of dried grasses by considering the ratio between brown and green grass leaf biomass (r_green_; Eq. (3); Fig. S4).

3 $${v}_{d}=\left\{\begin{array}{l}{v}_{d,\max }{\left(1-{e}^{\left(-0.0232{isi}\right)}\right)}^{1.6}0.7568{e}^{-17.8337\left(\frac{1}{{BUI}}-\frac{1}{64}\right)},{For\; Needleaf\; Trees}\\ {v}_{d,\max }{\left(1-{e}^{\left(-0.0282{isi}\right)}\right)}^{1.5}0.8482{e}^{-5.2680\left(\frac{1}{{BUI}}-\frac{1}{32}\right)},{For\; Broadleaf\; Trees}\\ {{{v}_{d,\max }r}_{{green}}\left(1-{e}^{\left(-0.031{isi}\right)}\right)}^{1.4}+\left(1.3{v}_{d,\max }\right){\left(1-{r}_{{green}}\right)\left(1-{e}^{\left(-0.035{isi}\right)}\right)}^{1.7},{For\; Grasses}\end{array}\right.$$

The v_d,max_ values derived from the Canadian FBP system^41^ and ref. ^29^ are 2.3 km h^−1^ for needleleaf evergreen and interior needleleaf evergreen trees, 3.8 km h^−1^ for continental needleleaf evergreen trees, 0.92 km h^−1^ for broadleaf cold deciduous trees, broadleaf evergreen shrubs, and broadleaf deciduous shrubs, and 4.97 km h^−1^ for C3 and C4 grasses.

The fire extinguishing probability, q, represents the probability that a fire will be extinguished on the same day it is initiated and is used to calculate the area burned for the fire event. The representation of q is algebraically reformulated based on the CLASSIC’s default fire module^20,65^ and Li et al.^74^. q is a function of population density, *p*_d_ (number of persons km^−2^), and increases from 0.5 at the minimum population density threshold *p*_d, min_ (number of persons km^−2^) to 1 at the maximum population density threshold *p*_d, max_ (Eq. (4); Fig. S4).

4 $$p=0.5+0.5\left(1-{e}^{\left(-\pi {\left(\frac{{p}_{d}}{{p}_{d,\mathrm{max}}}\right)}^{v}\right)}\right)$$

$$v=\frac{\mathrm{ln}\left(-\frac{\mathrm{ln}\left(1-{e}^{-\pi }\right)}{\pi }\right)}{n\left(\frac{{p}_{d,\mathrm{min}}}{{p}_{d,\mathrm{max}}}\right)}$$

*p*_d, min,_ and *p*_d, max_ are set at 0.001 and 0.1 based upon the 50th and 75th percentiles of population density within Canada’s managed forest fire response area^12^. This mathematical representation captures changes in future land use through the population density drivers (i.e., residential areas or farmland encroaching on forests). However, because the parameters *p*_d, min,_ *p*_d, max,_ and the dependence of human ignitions on population density are fixed they imply that fire management practices do not vary in time (Eq. (S1)).

The remaining calculations are unchanged compared to the CLASSIC’s default fire module^20,65^. The fire extinction probability determines the area burned over the duration of a fire, a_r,α_ (km^2^) for PFT α assuming an exponential distribution of fire duration based upon the area burned by a fire in one day (a_1,α_; Eq. (5)).

5 $${a}_{r,\alpha }={a}_{1,\alpha }\frac{(1-q)(2-q)}{{q}^{2}}$$

Finally, the area burned for each PFT α over the duration of the fire (A_b,α_; km^2^) is calculated as a function of the area of the grid cell (A_g_; km^2^), the fraction cover of PFT α (f_α_), a representative area (a_rep_; 800 km^2^), the probability of fire occurrence (*P*_f_), and the total area burned over the duration of the fire (a_r,α_; Eq. (6)).

6 $${A}_{b,\alpha }={P}_{f,\alpha }{a}_{r,\alpha }\frac{{A}_{g}{f}_{a}}{{a}_{{rep}}}$$

The calculation of fire-associated carbon (C) fluxes from vegetation biomass and litter remains unchanged as compared to the CLASSIC’s default fire module^20,65^. However, the FWI fire module has been modified compared to the CLASSIC’s default fire module to consider fire fluxes from the soil carbon pool. Carbon emissions to the atmosphere are calculated from each PFT burned area using pre-defined PFT-specific fire emission fractions for each live vegetation component (i.e., both structural and non-structural leaves, stems, and roots) as well as the litter pool (ʊ; Table S3)^65,77^. The PFT-specific fire emission fractions for soil account for the distribution of soil carbon within the soil profile, as well as its combustibility under average conditions^94–96^. The quantity of live vegetation carbon transferred to the litter pool as a result of fire-related mortality is calculated from each PFT burned area using pre-defined PFT-specific mortality fractions (ϴ; Table S3)^65,77^.

### Model forcing

CLASSIC requires seven meteorological forcing variables: incoming shortwave radiation, incoming longwave radiation, air temperature, precipitation rate, air pressure, specific humidity, and wind speed (see Table S1 for details of the simulations utilizing specific forcings). For model evaluation, we use a bias-corrected forcing based on reanalysis data (GSWP3–W5E5–ERA5), which is described in detail by Meyer et al.^91^ and Curasi et al.^22,77^. It combines the 1901–1978 portion of the Inter-Sectoral Impact Model Intercomparison Project (ISIMIP) GSWP3–W5E5 and the 1979–2018 portion of the ERA5 time series bias-corrected to match the means of the overlapping period in the GSWP3–W5E5^97–100^. This forcing is used alongside atmospheric CO_2_ concentrations (1700–2017) from the Global Carbon Project^101,102^ and population density from the Trends in the Land carbon cycle 2021 (TRENDY) protocol based on the History database of the Global Environment (Hyde) version 3.2^102,103^. For other analyses, we utilize climate forcings from the ISIMIP3b protocol for three Shared Socioeconomic Pathways (SSPs; SSP126, SSP370, and SSP585) and six Earth system models (i.e., the five primary ISIMIP models GFDL-ESM4, IPSL-CM6A-LR, MPI-ESM1-2-HR, MRI-ESM2-0, and UKESM1-0-LL along with CanESM5). We utilize model-specific bias-corrected historical 1850–2014 climate and model and SSP-specific future bias-corrected climate^43^. We disaggregated the climate forcing from daily to half-hourly time steps and linearly interpolated incoming longwave radiation following the methodology of Melton and Arora^65^ and nearest neighbor interpolated the 0.5° forcings to the 0.22° model grid. We utilized SSP-specific ISIMP3b Atmospheric CO_2_ concentrations (1850–2100)^104^ and ISIMP2b population density^75,105^.

The ISIMIP bias correction procedure utilizes different observational datasets than in GSWP3–W5E5–ERA5. They also represent average climate conditions and any intra-annual and interannual variations come from the underlying ESM output. As a result, modeled burned area increases with more limited agreement in interannual variation when compared to the NTEMS reference burned area (Fig. S5). Modeled mean fire CO_2_ emissions likewise increase slightly during the 2003–2015 reference period.

The FWI fire module requires cloud-to-ground lightning strike frequency. Therefore we derive a daily time series (1998–2021) of lightning strike frequency on the 0.22° model grid utilizing data from the Canadian Lightning Detection Network (CLDN)^68^. We apply multiplicative delta method bias correction^106^ to the time series utilizing climatological lightning from the Lightning Imaging Sensor Climatology and Optical Transient Detector Data Set (LISOTD)^107^. The result is a spatially and temporally explicit lightning strike frequency data set for the years 1998–2021. As a result of the bias correction, this data set has the same cumulative lightning strike frequency as the climatological lightning (LISOTD), which is utilized by CLASSIC’s default fire module globally, while preserving the detailed annual and interannual variability in CLDN. We derive two future (2022–2100) lightning strike frequencies for the three SSPs utilizing scenarios of global lightning density for the period 2014–2100 based on future climate simulations performed using UKESM1-0-LL^44^ and CanESM5^45^. We use the delta bias correction method for these scenarios based on the twelve years of overlap (2010–2021) between the projections and our historical lightning strike frequency time series. For UKESM1-0-LL lightning, we utilize multiplicative delta bias correction method and for CanESM5 lightning we utilize additive delta bias correction method^106^. We utilize the multiplicative delta method for datasets that incorporate terrestrial lightning observations (i.e., CLDN and UKESM1-0-LL) which can become saturated and have low detection efficiency during peak season events as compared to spaceborne observations (i.e., LISOTD)^108^. We utilize the additive delta method for CanESM5 lightning to account for the simulation’s spatial bias over eastern North America^45^.

In all of our simulations, the fractional coverage of PFTs is prescribed using the remotely sensed 14 PFT-hybrid land cover product generated by Wang et al.^109^ and expanded upon and evaluated by Curasi et al.^22^. This land cover corresponds to the year 2010 and does not vary in time.

### Simulation protocol

We carry out a total of seven historical simulations, 36 future simulations, and fifty-four factorial simulations using a common protocol (see Table S1 for details). We spin up the model allowing it to equilibrate carbon fluxes to conditions corresponding to the year 1850. We then perform transient historical simulations over the period from 1850 to 2014 and the future simulations over the period from 2015 to 2100 for each of the three SSPs. For the spin-up, we loop the earliest 26 years of model-specific bias-corrected climate data available (1850–1875, or 1901–1925) and 24 years of observed lightning strike frequencies (1998–2021), while holding atmospheric CO_2_ concentrations, and population density constant at the 1850 level. Then the transient historical run uses model-specific transient climate, transient atmospheric CO_2_ concentrations, and looping lightning strike frequencies (1998–2021) up until 1998 after which the observed lightning strike frequencies are used. The only exception is the simulation using the GSWP3–W5E5–ERA5 reanalysis where we utilize climatological mean daily lightning strike frequencies rather than looping observations before 1998 to illustrate lightning’s role in driving interannual variation in burned area. The 36 2015–2100 future simulations use model and SSP-specific bias-corrected climate projections, and SSP-specific transient atmospheric CO_2_ concentrations, population density, and SSP-specific lightning scenarios from two models (UKESM1-0-LL and CanESM5; see the model forcing section above). We use *t* tests to assess if the changes in burned area and fire CO_2_ emissions from the ensemble of forcings are spatially and temporally consistent. The fifty-four factorial simulations are intended to disentangle the contributions of CO_2_ fertilization (via its effect on vegetation biomass), climate trends, anthropogenic ignition/suppression (through population density) trends, and lightning ignition trends on the future changes in burned area and fire CO_2_ emissions. We did this by conducting model and SSP-specific future simulations with different combinations of these forcings fixed at their 2014 levels to isolate their impact on the simulated trends following the methods of Kou-Giesbrecht and Arora^76^. These simulations are detailed in Table S1. We conduct further sensitivity analysis, by plotting the relationships between mean annual temperature, lightning strike frequency, vegetation carbon, precipitation, population density, burned area, and fire CO_2_ emissions (Figs. 3, S2, and S3). This analysis utilized the annual values from all the future and factorial simulations (90 simulations from 2014–2100; *n* = 7740 points visualized).

### Model evaluation datasets

We evaluated the CLASSIC fire module’s historical performance against an ensemble of observation-based burned area and fire CO_2_ emissions datasets (see Table 1 for an overview). We include a large number of reference datasets to quantify the uncertainty in the reference datasets themselves^22,110^. As a result, once the model falls within the range of the reference datasets improvements in performance against any one data set lead to a deterioration in performance against others^22,110^. The remotely sensed burned area reference datasets come from the Global Fire Emissions Database version 4.1 with small fires (GFED4.1 s)^111^, European Space Agency Climate Change Initiative FireCCI50 MODIS derived burned area (MODIS)^112^, and the National Terrestrial Ecosystem Monitoring System for Canada (NTEMS)^113,114^. The fire CO_2_ emissions datasets come from several sources including the Global Fire Emissions Database version 4.1 with small fires (GFED4.1 s), the Fire Inventory from NCAR version 2.5 (FINN2.5)^55^, Fire Energetics and Emissions Research version 1.0-G1.2 (FEER1.0-G1.2) (available from http://feer.gsfc.nasa.gov/data/emissions/)^57^, the Quick Fire Emissions Dataset version 2.4 revision 1 (QFED2.4r1) (available from https://portal.nccs.nasa.gov/datashare/iesa/aerosol/emissions/QFED/v2.4r6/)^58^, and Carbon Tracker 2019 (CT2019) (available from https://gml.noaa.gov/aftp/products/carbontracker/co2/)^53,54^. All model summary and plotting operations were carried out in R^115^.

## Supplementary information

Supplementary Information

## Data Availability

The model evaluation datasets herein are open-access and publicly available via their respective references. The model outputs that support this publication are archived on Zenodo (10.5281/zenodo.13799947).

## Code availability

The CLASSIC source code that supports this publication is archived on Zenodo (10.5281/zenodo.13799947).
