# Supplementary material for: Global climate change below 2 °C avoids large end century increases in burned area in Canada
Source: NPJ Clim Atmos Sci. 2024 Oct 1;7(1):228. doi: 10.1038/s41612-024-00781-4 (PMC11442306; doi:10.1038/s41612-024-00781-4)
Supplement: Supplementary file 1 — Supplementary Information [file 41612_2024_781_MOESM1_ESM.pdf]

Supplementary materials for: Global climate change below 2°C avoids large end century increases in burned area in Canada

Salvatore R. Curasi[1,2,3,https://orcid.org/0000-0002-4534-3344], Joe R. Melton[2,https://orcid.org/0000-0002-9414-064X], Vivek K. Arora[1,https://orcid.org/0000-0002-2620-9342], Elyn R. Humphreys[3,https://orcid.org/0000-0002-5397-2802], and Cynthia Whaley[1,https://orcid.org/0000-0002-0028-1514]

[1]Canadian Centre for Climate Modelling and Analysis, Environment and Climate Change Canada, Victoria, BC, Canada
[2]Climate Research Division, Environment, and Climate Change Canada, Victoria, BC, Canada
[3]Department of Geography & Environmental Studies, Carleton University, Ottawa, ON, Canada

Supplementary figures:

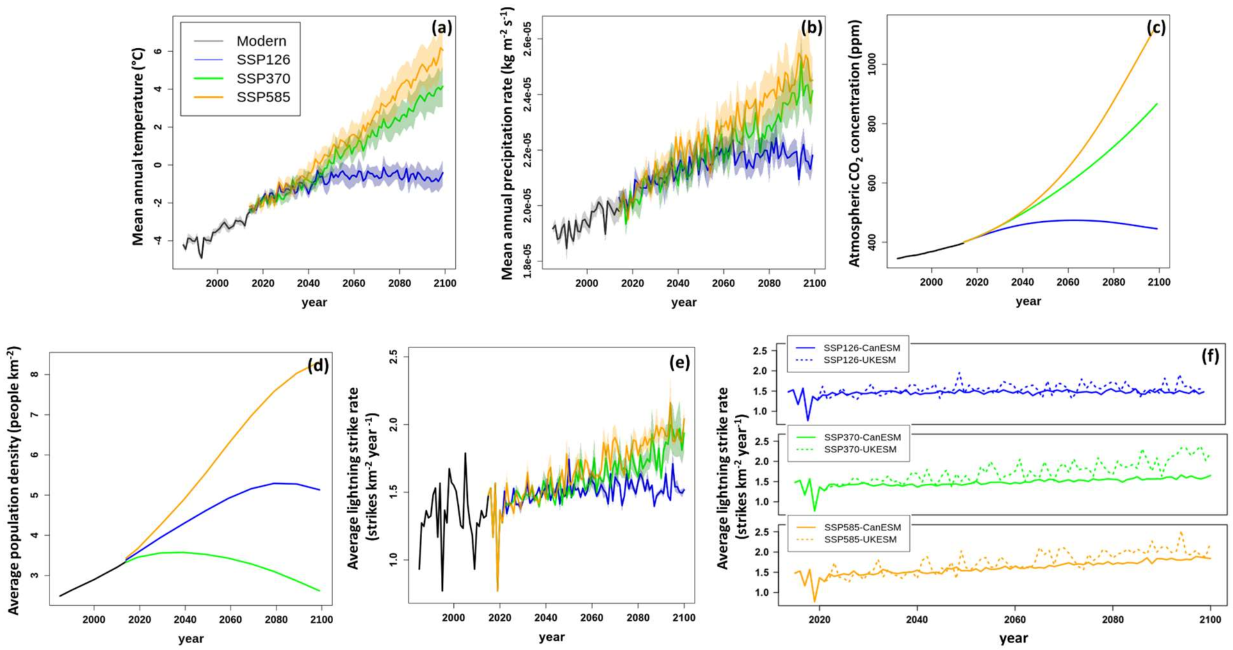

Figure S1: Canada-wide mean summary plots for drivers utilized by the model including **a)** mean annual temperature **b)** mean annual precipitation rate, **c)** atmospheric CO_2 concentration, **d)** average population density, **e)** average lightning strike rate, and **f)** average lightning strike rate broken down by future driver source and SSP. The shaded regions denote one standard error around the mean for the ensemble of six earth system model projections in “a” and “b” and two lightning model projections in “e”.

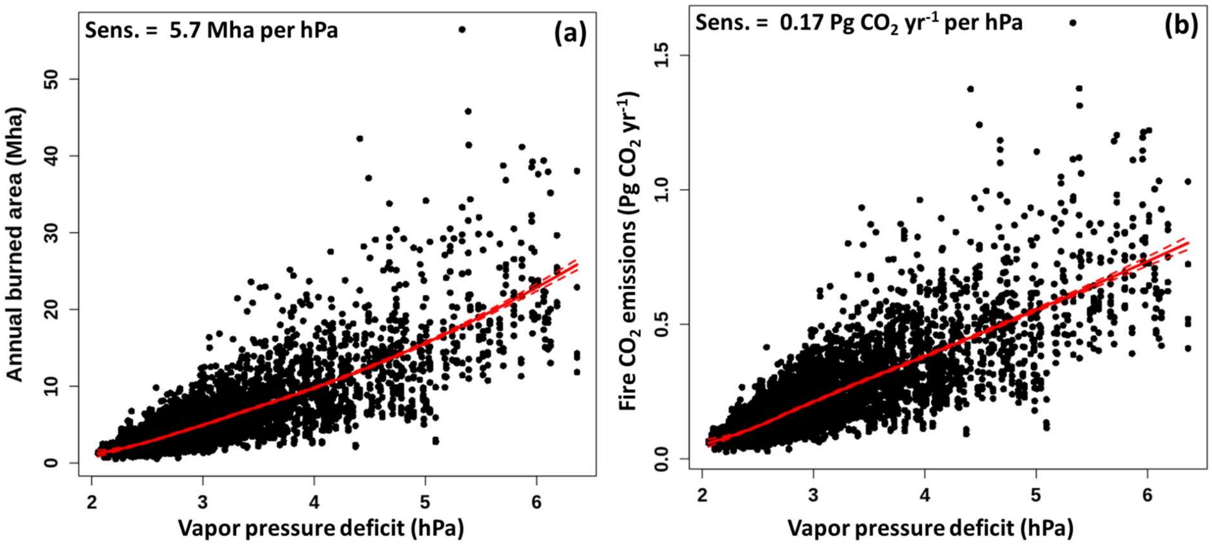

Figure S2: Sensitivity plots of the relationship between Canada-wide mean annual vapor pressure deficit **a)** annual burned area, and **b)** Fire CO_2 emissions. The plot visualizes all 90 simulations from 2014 - 2100 (n = 7740 points visualized) and includes a LOESS smoothed regression line in red with 95% confidence intervals.

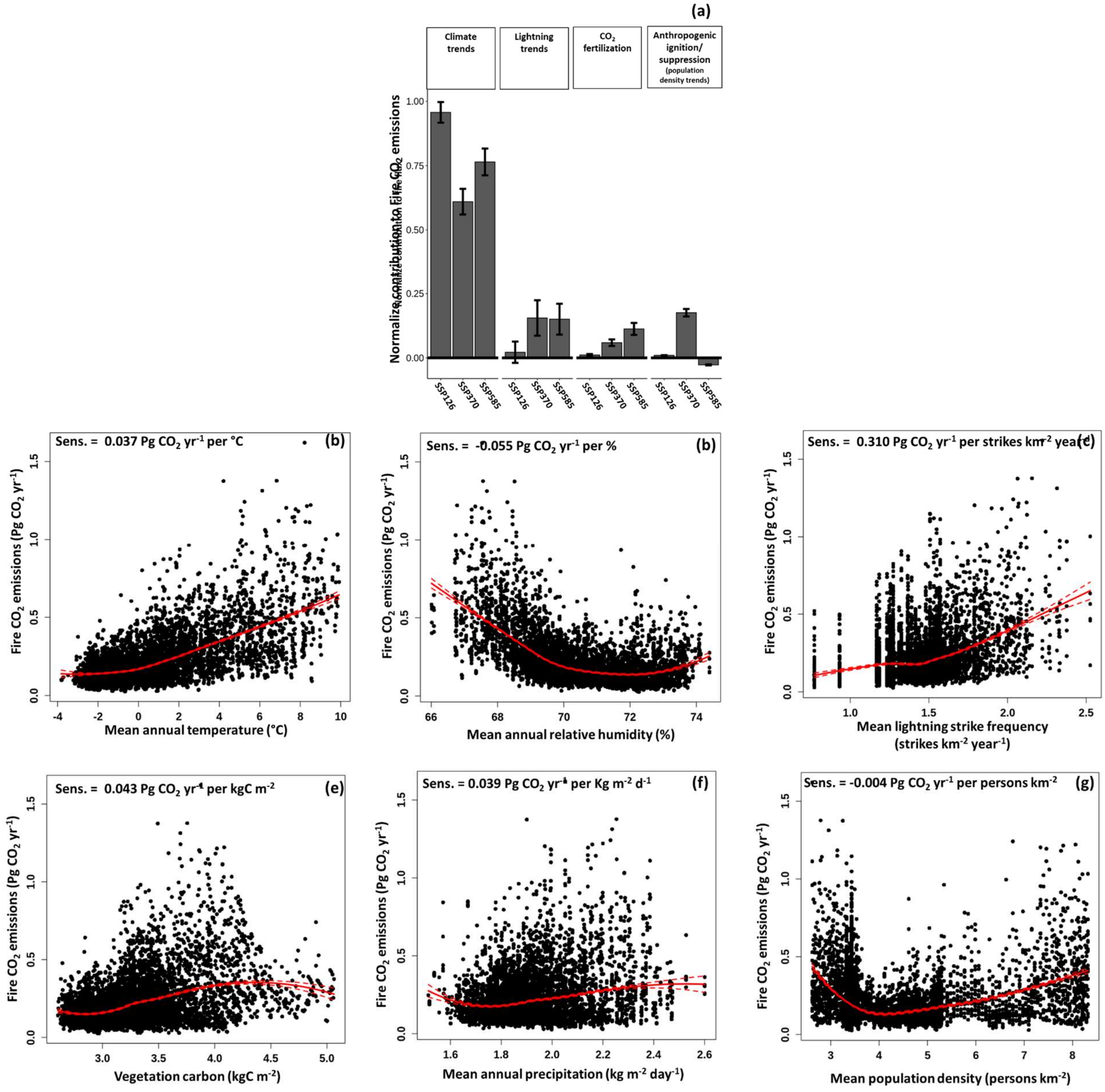

Figure S3: a) The contribution of climate trends, lightning trends, CO_2 fertilization, and anthropogenic ignition/suppression (population density) to fire CO_2 emissions normalized from 0 (0% of the trend) to 1 (100% of the trend). As well as the dependence between Fire CO_2 emissions and a) **b)** Canada-wide mean annual temperature, **c)** Canada-wide mean annual lightning strike frequency, **d)** Canada-wide mean annual vegetation carbon, **e)** Canada-wide mean annual precipitation and **f)** Canada-wide mean annual population density. The plots visualize all 90 simulations from 2014 - 2100 (n = 7740 points visualized) and include a LOESS smoothed regression line in red with 95% confidence intervals.

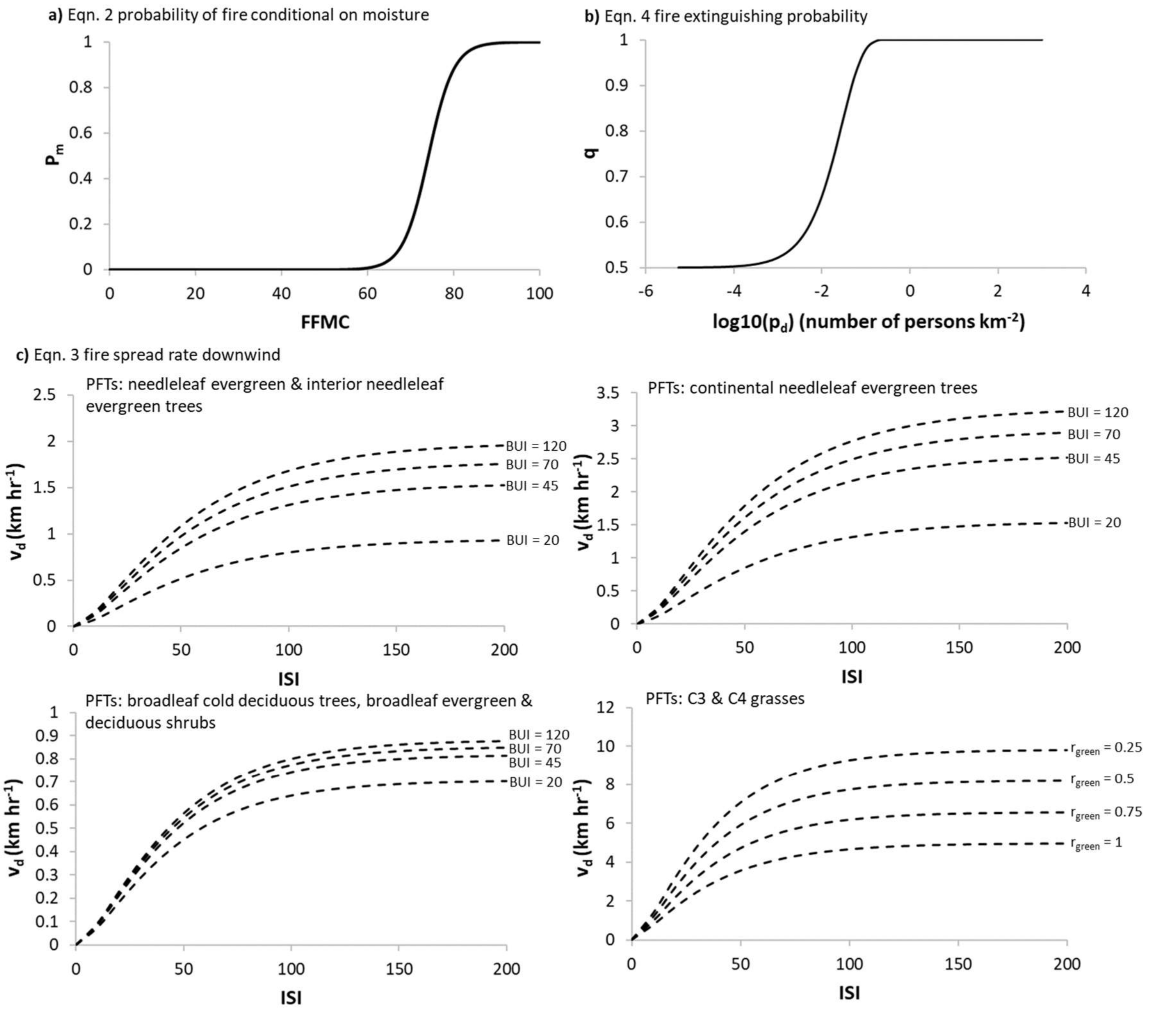

Figure S4: Contour plots of **a)** the probability of fire conditional on moisture (P_m; Eqn. 2) as a function of the fine fuel moisture code (FFMC), **b)** the fire extinguishing probability (q; Eqn. 4) as a function of population density (p_d), and **c)** the fire spread rates in the downwind direction (V_d; Eqn. 3) for all burnable boreal plant functional types (PFTs) a function of the initial spread index (ISI) and the build up index (BUI), or in the case of the ratio between brown and green grass leaf biomass (r_{green}).

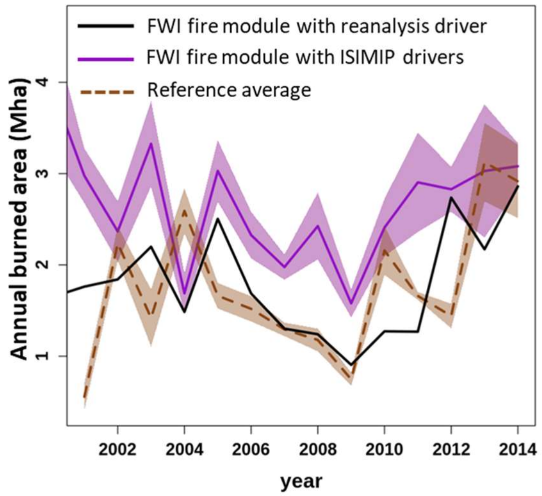

Figure S5: Comparison between the FWI fire module driven by six Inter-Sectoral Impact Model Intercomparison Project (ISIMIP) bias-corrected Earth system model outputs, reanalysis climate (GSWP3–W5E5–ERA5), and the mean of the three burned area reference data sets. For the FWI fire module with ISIMIP drivers burned area = 2.57 ± 0.5 Mha (2001 - 2015) and Pearson r = 0.16.

Supplementary tables:

Table S1: Simulations carried out and evaluated herein. The acronyms used herein are detailed within the methods.

#	Type	Years	In sensitivity[1]	ISIMIP climate forcing[2]	Atmospheric CO_2[3]	Population density[4]	lightning strike frequency[5]
1	Historical	1850 - 2017	No	Reanalysis (GSWP3–W5E5–ERA5)	global carbon project	TRENDY-Hyde	CLDN fuse w/ LISODT (climatological lightning before 1998)
2	Historical	1850 - 2014	No	GFDL-ESM4	ISIMP3b	ISIMP2b	CLDN fuse w/ LISODT (1998 - 2021 looped before 1998)
3	Historical	1850 - 2014	No	IPSL-CM6A-LR	ISIMP3b	ISIMP2b	CLDN fuse w/ LISODT (1998 - 2021 looped before 1998)
4	Historical	1850 - 2014	No	MPI-ESM1-2-HR	ISIMP3b	ISIMP2b	CLDN fuse w/ LISODT (1998 - 2021 looped before 1998)
5	Historical	1850 - 2014	No	MRI-ESM2-0	ISIMP3b	ISIMP2b	CLDN fuse w/ LISODT (1998 - 2021 looped before 1998)
6	Historical	1850 - 2014	No	UKESM1-0-LL	ISIMP3b	ISIMP2b	CLDN fuse w/ LISODT (1998 - 2021 looped before 1998)
7	Historical	1850 - 2014	No	CanESM5	ISIMP3b	ISIMP2b	CLDN fuse w/ LISODT (1998 - 2021 looped before 1998)
8	Future	2015 - 2100	Yes	GFDL-ESM4 (SSP126)	ISIMP3b	ISIMP2b	projected w/ UKESM1-0-LL
9	Future	2015 - 2100	Yes	IPSL-CM6A-LR (SSP126)	ISIMP3b	ISIMP2b	projected w/ UKESM1-0-LL
10	Future	2015 - 2100	Yes	MPI-ESM1-2-HR (SSP126)	ISIMP3b	ISIMP2b	projected w/ UKESM1-0-LL
11	Future	2015 - 2100	Yes	MRI-ESM2-0 (SSP126)	ISIMP3b	ISIMP2b	projected w/ UKESM1-0-LL
12	Future	2015 - 2100	Yes	UKESM1-0-LL (SSP126)	ISIMP3b	ISIMP2b	projected w/ UKESM1-0-LL
13	Future	2015 - 2100	Yes	CanESM5 (SSP126)	ISIMP3b	ISIMP2b	projected w/ UKESM1-0-LL
14	Future	2015 - 2100	Yes	GFDL-ESM4 (SSP370)	ISIMP3b	ISIMP2b	projected w/ UKESM1-0-LL
15	Future	2015 - 2100	Yes	IPSL-CM6A-LR (SSP370)	ISIMP3b	ISIMP2b	projected w/ UKESM1-0-LL
16	Future	2015 - 2100	Yes	MPI-ESM1-2-HR (SSP370)	ISIMP3b	ISIMP2b	projected w/ UKESM1-0-LL
17	Future	2015 - 2100	Yes	MRI-ESM2-0 (SSP370)	ISIMP3b	ISIMP2b	projected w/ UKESM1-0-LL
18	Future	2015 - 2100	Yes	UKESM1-0-LL (SSP370)	ISIMP3b	ISIMP2b	projected w/ UKESM1-0-LL
19	Future	2015 - 2100	Yes	CanESM5 (SSP370)	ISIMP3b	ISIMP2b	projected w/ UKESM1-0-LL
20	Future	2015 - 2100	Yes	GFDL-ESM4 (SSP585)	ISIMP3b	ISIMP2b	projected w/ UKESM1-0-LL
21	Future	2015 - 2100	Yes	IPSL-CM6A-LR (SSP585)	ISIMP3b	ISIMP2b	projected w/ UKESM1-0-LL
22	Future	2015 - 2100	Yes	MPI-ESM1-2-HR (SSP585)	ISIMP3b	ISIMP2b	projected w/ UKESM1-0-LL
23	Future	2015 - 2100	Yes	MRI-ESM2-0 (SSP585)	ISIMP3b	ISIMP2b	projected w/ UKESM1-0-LL
24	Future	2015 - 2100	Yes	UKESM1-0-LL (SSP585)	ISIMP3b	ISIMP2b	projected w/ UKESM1-0-LL
25	Future	2015 - 2100	Yes	CanESM5 (SSP585)	ISIMP3b	ISIMP2b	projected w/ UKESM1-0-LL
26	Future	2015 - 2100	Yes	GFDL-ESM4 (SSP126)	ISIMP3b	ISIMP2b	projected w/ CanESM5
27	Future	2015 - 2100	Yes	IPSL-CM6A-LR (SSP126)	ISIMP3b	ISIMP2b	projected w/ CanESM5
28	Future	2015 - 2100	Yes	MPI-ESM1-2-HR (SSP126)	ISIMP3b	ISIMP2b	projected w/ CanESM5
29	Future	2015 - 2100	Yes	MRI-ESM2-0 (SSP126)	ISIMP3b	ISIMP2b	projected w/ CanESM5
30	Future	2015 - 2100	Yes	UKESM1-0-LL (SSP126)	ISIMP3b	ISIMP2b	projected w/ CanESM5
31	Future	2015 - 2100	Yes	CanESM5 (SSP126)	ISIMP3b	ISIMP2b	projected w/ CanESM5
32	Future	2015 - 2100	Yes	GFDL-ESM4 (SSP370)	ISIMP3b	ISIMP2b	projected w/ CanESM5
33	Future	2015 - 2100	Yes	IPSL-CM6A-LR (SSP370)	ISIMP3b	ISIMP2b	projected w/ CanESM5
34	Future	2015 - 2100	Yes	MPI-ESM1-2-HR (SSP370)	ISIMP3b	ISIMP2b	projected w/ CanESM5
35	Future	2015 - 2100	Yes	MRI-ESM2-0 (SSP370)	ISIMP3b	ISIMP2b	projected w/ CanESM5
36	Future	2015 - 2100	Yes	UKESM1-0-LL (SSP370)	ISIMP3b	ISIMP2b	projected w/ CanESM5
37	Future	2015 - 2100	Yes	CanESM5 (SSP370)	ISIMP3b	ISIMP2b	projected w/ CanESM5
38	Future	2015 - 2100	Yes	GFDL-ESM4 (SSP585)	ISIMP3b	ISIMP2b	projected w/ CanESM5
39	Future	2015 - 2100	Yes	IPSL-CM6A-LR (SSP585)	ISIMP3b	ISIMP2b	projected w/ CanESM5
40	Future	2015 - 2100	Yes	MPI-ESM1-2-HR (SSP585)	ISIMP3b	ISIMP2b	projected w/ CanESM5
41	Future	2015 - 2100	Yes	MRI-ESM2-0 (SSP585)	ISIMP3b	ISIMP2b	projected w/ CanESM5
42	Future	2015 - 2100	Yes	UKESM1-0-LL (SSP585)	ISIMP3b	ISIMP2b	projected w/ CanESM5
43	Future	2015 - 2100	Yes	CanESM5 (SSP585)	ISIMP3b	ISIMP2b	projected w/ CanESM5
44	Factorial	2015 - 2100	Yes	GFDL-ESM4 (SSP126)	ISIMP3b (2014 constant)	ISIMP2b (2014 constant)	CLDN fuse w/ LISODT (1998 - 2021 looped)
45	Factorial	2015 - 2100	Yes	IPSL-CM6A-LR (SSP126)	ISIMP3b (2014 constant)	ISIMP2b (2014 constant)	CLDN fuse w/ LISODT (1998 - 2021 looped)
46	Factorial	2015 - 2100	Yes	MPI-ESM1-2-HR (SSP126)	ISIMP3b (2014 constant)	ISIMP2b (2014 constant)	CLDN fuse w/ LISODT (1998 - 2021 looped)
47	Factorial	2015 - 2100	Yes	MRI-ESM2-0 (SSP126)	ISIMP3b (2014 constant)	ISIMP2b (2014 constant)	CLDN fuse w/ LISODT (1998 - 2021 looped)
48	Factorial	2015 - 2100	Yes	UKESM1-0-LL (SSP126)	ISIMP3b (2014 constant)	ISIMP2b (2014 constant)	CLDN fuse w/ LISODT (1998 - 2021 looped)
49	Factorial	2015 - 2100	Yes	CanESM5 (SSP126)	ISIMP3b (2014 constant)	ISIMP2b (2014 constant)	CLDN fuse w/ LISODT (1998 - 2021 looped)
50	Factorial	2015 - 2100	Yes	GFDL-ESM4 (SSP126)	ISIMP3b	ISIMP2b (2014 constant)	CLDN fuse w/ LISODT (1998 - 2021 looped)
51	Factorial	2015 - 2100	Yes	IPSL-CM6A-LR (SSP126)	ISIMP3b	ISIMP2b (2014 constant)	CLDN fuse w/ LISODT (1998 - 2021 looped)
52	Factorial	2015 - 2100	Yes	MPI-ESM1-2-HR (SSP126)	ISIMP3b	ISIMP2b (2014 constant)	CLDN fuse w/ LISODT (1998 - 2021 looped)
53	Factorial	2015 - 2100	Yes	MRI-ESM2-0 (SSP126)	ISIMP3b	ISIMP2b (2014 constant)	CLDN fuse w/ LISODT (1998 - 2021 looped)
54	Factorial	2015 - 2100	Yes	UKESM1-0-LL (SSP126)	ISIMP3b	ISIMP2b (2014 constant)	CLDN fuse w/ LISODT (1998 - 2021 looped)
55	Factorial	2015 - 2100	Yes	CanESM5 (SSP126)	ISIMP3b	ISIMP2b (2014 constant)	CLDN fuse w/ LISODT (1998 - 2021 looped)
56	Factorial	2015 - 2100	Yes	GFDL-ESM4 (SSP126)	ISIMP3b	ISIMP2b	CLDN fuse w/ LISODT (1998 - 2021 looped)
57	Factorial	2015 - 2100	Yes	IPSL-CM6A-LR (SSP126)	ISIMP3b	ISIMP2b	CLDN fuse w/ LISODT (1998 - 2021 looped)
58	Factorial	2015 - 2100	Yes	MPI-ESM1-2-HR (SSP126)	ISIMP3b	ISIMP2b	CLDN fuse w/ LISODT (1998 - 2021 looped)
59	Factorial	2015 - 2100	Yes	MRI-ESM2-0 (SSP126)	ISIMP3b	ISIMP2b	CLDN fuse w/ LISODT (1998 - 2021 looped)
60	Factorial	2015 - 2100	Yes	UKESM1-0-LL (SSP126)	ISIMP3b	ISIMP2b	CLDN fuse w/ LISODT (1998 - 2021 looped)
61	Factorial	2015 - 2100	Yes	CanESM5 (SSP126)	ISIMP3b	ISIMP2b	CLDN fuse w/ LISODT (1998 - 2021 looped)
62	Factorial	2015 - 2100	Yes	GFDL-ESM4 (SSP370)	ISIMP3b (2014 constant)	ISIMP2b (2014 constant)	CLDN fuse w/ LISODT (1998 - 2021 looped)
63	Factorial	2015 - 2100	Yes	IPSL-CM6A-LR (SSP370)	ISIMP3b (2014 constant)	ISIMP2b (2014 constant)	CLDN fuse w/ LISODT (1998 - 2021 looped)
64	Factorial	2015 - 2100	Yes	MPI-ESM1-2-HR (SSP370)	ISIMP3b (2014 constant)	ISIMP2b (2014 constant)	CLDN fuse w/ LISODT (1998 - 2021 looped)
65	Factorial	2015 - 2100	Yes	MRI-ESM2-0 (SSP370)	ISIMP3b (2014 constant)	ISIMP2b (2014 constant)	CLDN fuse w/ LISODT (1998 - 2021 looped)
66	Factorial	2015 - 2100	Yes	UKESM1-0-LL (SSP370)	ISIMP3b (2014 constant)	ISIMP2b (2014 constant)	CLDN fuse w/ LISODT (1998 - 2021 looped)
67	Factorial	2015 - 2100	Yes	CanESM5 (SSP370)	ISIMP3b (2014 constant)	ISIMP2b (2014 constant)	CLDN fuse w/ LISODT (1998 - 2021 looped)
68	Factorial	2015 - 2100	Yes	GFDL-ESM4 (SSP370)	ISIMP3b	ISIMP2b (2014 constant)	CLDN fuse w/ LISODT (1998 - 2021 looped)

69	Factorial	2015 - 2100	Yes	IPSL-CM6A-LR (SSP370)	ISIMIP3b	ISIMIP2b (2014 constant)	CLDN fuse w/ LISODT (1998 - 2021 looped)
70	Factorial	2015 - 2100	Yes	MPI-ESM1-2-HR (SSP370)	ISIMIP3b	ISIMIP2b (2014 constant)	CLDN fuse w/ LISODT (1998 - 2021 looped)
71	Factorial	2015 - 2100	Yes	MRI-ESM2-0 (SSP370)	ISIMIP3b	ISIMIP2b (2014 constant)	CLDN fuse w/ LISODT (1998 - 2021 looped)
72	Factorial	2015 - 2100	Yes	UKESM1-0-LL (SSP370)	ISIMIP3b	ISIMIP2b (2014 constant)	CLDN fuse w/ LISODT (1998 - 2021 looped)
73	Factorial	2015 - 2100	Yes	CanESM5 (SSP370)	ISIMIP3b	ISIMIP2b (2014 constant)	CLDN fuse w/ LISODT (1998 - 2021 looped)
74	Factorial	2015 - 2100	Yes	GFDL-ESM4 (SSP370)	ISIMIP3b	ISIMIP2b	CLDN fuse w/ LISODT (1998 - 2021 looped)
75	Factorial	2015 - 2100	Yes	IPSL-CM6A-LR (SSP370)	ISIMIP3b	ISIMIP2b	CLDN fuse w/ LISODT (1998 - 2021 looped)
76	Factorial	2015 - 2100	Yes	MPI-ESM1-2-HR (SSP370)	ISIMIP3b	ISIMIP2b	CLDN fuse w/ LISODT (1998 - 2021 looped)
77	Factorial	2015 - 2100	Yes	MRI-ESM2-0 (SSP370)	ISIMIP3b	ISIMIP2b	CLDN fuse w/ LISODT (1998 - 2021 looped)
78	Factorial	2015 - 2100	Yes	UKESM1-0-LL (SSP370)	ISIMIP3b	ISIMIP2b	CLDN fuse w/ LISODT (1998 - 2021 looped)
79	Factorial	2015 - 2100	Yes	CanESM5 (SSP370)	ISIMIP3b	ISIMIP2b	CLDN fuse w/ LISODT (1998 - 2021 looped)
80	Factorial	2015 - 2100	Yes	GFDL-ESM4 (SSP585)	ISIMIP3b (2014 constant)	ISIMIP2b (2014 constant)	CLDN fuse w/ LISODT (1998 - 2021 looped)
81	Factorial	2015 - 2100	Yes	IPSL-CM6A-LR (SSP585)	ISIMIP3b (2014 constant)	ISIMIP2b (2014 constant)	CLDN fuse w/ LISODT (1998 - 2021 looped)
82	Factorial	2015 - 2100	Yes	MPI-ESM1-2-HR (SSP585)	ISIMIP3b (2014 constant)	ISIMIP2b (2014 constant)	CLDN fuse w/ LISODT (1998 - 2021 looped)
83	Factorial	2015 - 2100	Yes	MRI-ESM2-0 (SSP585)	ISIMIP3b (2014 constant)	ISIMIP2b (2014 constant)	CLDN fuse w/ LISODT (1998 - 2021 looped)
84	Factorial	2015 - 2100	Yes	UKESM1-0-LL (SSP585)	ISIMIP3b (2014 constant)	ISIMIP2b (2014 constant)	CLDN fuse w/ LISODT (1998 - 2021 looped)
85	Factorial	2015 - 2100	Yes	CanESM5 (SSP585)	ISIMIP3b (2014 constant)	ISIMIP2b (2014 constant)	CLDN fuse w/ LISODT (1998 - 2021 looped)
86	Factorial	2015 - 2100	Yes	GFDL-ESM4 (SSP585)	ISIMIP3b	ISIMIP2b (2014 constant)	CLDN fuse w/ LISODT (1998 - 2021 looped)
87	Factorial	2015 - 2100	Yes	IPSL-CM6A-LR (SSP585)	ISIMIP3b	ISIMIP2b (2014 constant)	CLDN fuse w/ LISODT (1998 - 2021 looped)
88	Factorial	2015 - 2100	Yes	MPI-ESM1-2-HR (SSP585)	ISIMIP3b	ISIMIP2b (2014 constant)	CLDN fuse w/ LISODT (1998 - 2021 looped)
89	Factorial	2015 - 2100	Yes	MRI-ESM2-0 (SSP585)	ISIMIP3b	ISIMIP2b (2014 constant)	CLDN fuse w/ LISODT (1998 - 2021 looped)
90	Factorial	2015 - 2100	Yes	UKESM1-0-LL (SSP585)	ISIMIP3b	ISIMIP2b (2014 constant)	CLDN fuse w/ LISODT (1998 - 2021 looped)
91	Factorial	2015 - 2100	Yes	CanESM5 (SSP585)	ISIMIP3b	ISIMIP2b (2014 constant)	CLDN fuse w/ LISODT (1998 - 2021 looped)
92	Factorial	2015 - 2100	Yes	GFDL-ESM4 (SSP585)	ISIMIP3b	ISIMIP2b	CLDN fuse w/ LISODT (1998 - 2021 looped)
93	Factorial	2015 - 2100	Yes	IPSL-CM6A-LR (SSP585)	ISIMIP3b	ISIMIP2b	CLDN fuse w/ LISODT (1998 - 2021 looped)
94	Factorial	2015 - 2100	Yes	MPI-ESM1-2-HR (SSP585)	ISIMIP3b	ISIMIP2b	CLDN fuse w/ LISODT (1998 - 2021 looped)
95	Factorial	2015 - 2100	Yes	MRI-ESM2-0 (SSP585)	ISIMIP3b	ISIMIP2b	CLDN fuse w/ LISODT (1998 - 2021 looped)
96	Factorial	2015 - 2100	Yes	UKESM1-0-LL (SSP585)	ISIMIP3b	ISIMIP2b	CLDN fuse w/ LISODT (1998 - 2021 looped)
97	Factorial	2015 - 2100	Yes	CanESM5 (SSP585)	ISIMIP3b	ISIMIP2b	CLDN fuse w/ LISODT (1998 - 2021 looped)

[1]Denotes runs included in the figure 3b-f, S2, and S3b-f sensitivity analysis.

[2]For details of the GSWP3–W5E5–ERA5 forcing see Meyer et al.[1] and Curasi et al.[2,3], and for details of the ISIMIP climate forcings see Lange and Buchner[4].

[3]For details of the global carbon project atmospheric CO_2 concentrations see Friedlingstein et al.[5], and for details of the ISIMIP3b atmospheric CO_2 concentrations see Buchner and Reyer[6].

[4]For details of the TRENDY-Hyde population density data see Chini et al.[7] and Friedlingstein et al.[5], and for details of the ISIMIP2b population density see Klein Goldewijk et al.[8] and Jones and O'Neill[9].

[5]For details of the CLDN, LISOTD, UKESM, and CanESM5 lightning datasets see Burrows and Kochtubajda[10], Cecil et al.[11], Kaplan et al.[12], and Whaley et al.[13] respectively.

Table S2: Canada-wide Pearson correlations for mean annual values of atmospheric drivers that influence the fire weather index (FWI), during the historical period (1985 - 2014), based upon reanalysis (GSWP3–W5E5–ERA5).

Variable	Specific humidity	Wind speed	Temperature
Specific humidity	_		
Wind speed	-0.45	_	
Temperature	0.91	-0.47	_
Precipitation	0.31	0.25	0.24

Table S3: Fire emission fractions (ʊ) for various classifications of PFTs used to calculate carbon emissions to the atmosphere due to fire for each live vegetation component (i.e. both structural and non-structural leaves, stems, and roots), the litter pool, and the soil pool. As well as the PFT-specific mortality fractions (Ɵ) used to calculate the quantity of carbon from each live vegetation component transferred to the litter pool. Trees PFTs include Needleleaf evergreen, Needleleaf deciduous, Continental needleleaf evergreen, Interior needleleaf evergreen, Broadleaf evergreen, Broadleaf cold deciduous, and Broadleaf drought/dry deciduous trees. Herbaceous PFTs include C3 grass, C4 grass, and Sedge PFTs. Shrub PFTs include Broadleaf evergreen and Broadleaf deciduous shrubs (See Curasi et al.[2] for further details). Crop PFTs are not impacted by fire and therefore not assigned fractions.

	green leaves		brown leaves		stems		roots		litter	soil
PFT type	combusted (ʊL)	litter (ƟL)	combusted (ʊB)	litter (ƟB)	combusted (ʊS)	litter (ƟS)	combusted (ʊR)	litter (ƟR)	combusted (ʊD)	combusted (ʊS)
Tree	0.42	0.20	-	-	0.12	0.60	0.00	0.10	0.30	0.030
Herbaceous	0.48	0.10	0.54	0.06	0.00	0.00	0.00	0.25	0.42	0.042
Shrub	0.42	0.20	-	-	0.12	0.60	0.00	0.10	0.36	0.036

Supplementary equations

Equations S1:

As detailed in Metlon et al.[14] The probability of fire conditional on ignition (P_i) is the cumulative contribution of both natural lightning-driven ($P_{i,n}$) and anthropogenic ($P_{i,a}$) ignition sources (S1.1).

$$P_i = max\left[0, \min(1, P_{i,n} + [1 - P_{i,n}]P_{i,a})\right] \quad \text{(S1.1)}$$

$P_{i,n}$ varies from 0 to 1 depending upon the number of cloud-to-ground lightning strikes in a particular location (F_{c2g}; strikes km^{-2} year^{-1}) between upper and lower thresholds of 0.25 and 10 strikes km^{-2} year^{-1}.

$$v_f = max\left[0, \min\left(1, \frac{F_{c2g} - 0.25}{10 - 0.25}\right)\right]$$

$$y(v_f) = \frac{1}{1 + \exp\left(\frac{0.8 - v_f}{0.1}\right)}$$

$$P_{i,n} = y(v_f) - y(0)(1 - v_f) + v_f[1 - y(1)] \quad \text{(S1.2)}$$

$P_{i,a}$ is dependent upon population density (p_d; number of persons km^{-2}) following from Kloster et al.[15] and approaches one at an upper population density threshold of 300 people km^{-2}.

$$P_{i,a} = min\left[1, \left(\frac{P_d}{300}\right)\right] \quad \text{(S1.3)}$$

References:

1. Meyer, G., Humphreys, E. R., Melton, J. R., Cannon, A. J. & Lafleur, P. M. Simulating shrubs and their energy and carbon dioxide fluxes in Canada's Low Arctic with the Canadian Land Surface Scheme Including Biogeochemical Cycles (CLASSIC). *Biogeosciences* **18**, 3263–3283 (2021).

2. Curasi, S. R. *et al.* Evaluating the performance of the Canadian Land Surface Scheme Including Biogeochemical Cycles (CLASSIC) tailored to the pan-Canadian domain. *Journal of Advances in Modeling Earth Systems* **15**, e2022MS003480 (2023).

3. Curasi, S. R., Melton, J. R. & Humphreys, E. R. Implementing a dynamic representation of fire and harvest including subgrid-scale heterogeneity in the tile-based land surface model CLASSIC v1. 45. *Geoscientific Model Development* **17**, 2683–2704 (2024).

4. Lange, S. & Büchner, M. ISIMIP3b bias-adjusted atmospheric climate input data (v1. 1). (2021).

5. Friedlingstein, P. *et al.* Global carbon budget 2021. *Earth System Science Data* **14**, 1917–2005 (2022).

6. Büchner, M. & Reyer, C. ISIMIP3b atmospheric composition input data. ISIMIP

Repository https://doi.org/10.48364/ISIMIP.482153.1 (2022).

7. Chini, L. *et al.* Land-use harmonization datasets for annual global carbon budgets. *Earth Syst. Sci. Data* **13**, 4175–4189 (2021).

8. Klein Goldewijk, K., Beusen, A., Doelman, J. & Stehfest, E. Anthropogenic land use estimates for the Holocene – HYDE 3.2. *Earth Syst. Sci. Data* **9**, 927–953 (2017).

9. Jones, B. & O'Neill, B. C. Spatially explicit global population scenarios consistent with the Shared Socioeconomic Pathways. *Environ. Res. Lett.* **11**, 1–10 (2016).

10. Burrows, W. R. & Kochtubajda, B. A decade of cloud-to-ground lightning in Canada: 1999–2008. Part 1: Flash density and occurrence. *Atmosphere-Ocean* **48**, 177–194 (2010).

11. Cecil, Buechler & Blakeslee. LIS/OTD Gridded Lightning Climatology Data Sets. . *gov/pub/lis/climatology/LIS-OTD/HRFC/] from the …* (2014).

12. Kaplan, J. O., Koch, A. & Lau, K. H.-K. *Estimated Future Global Lightning Strokes (2010-2100)*. (2023). doi:10.5281/zenodo.7511843.

13. Whaley, C. *et al.* A new lightning scheme in Canada's Atmospheric Model, CanAM5.1: Implementation, evaluation, and projections of lightning and fire in future climates. *Geoscientific Model Development Discussion* (2024) doi:10.5194/gmd-2024-24.

14. Melton, J. R. & Arora, V. K. Competition between plant functional types in the Canadian Terrestrial Ecosystem Model (CTEM) v. 2.0. *Geosci. Model Dev.* **9**, 323–361 (2016).

15. Kloster, S. *et al.* Fire dynamics during the 20th century simulated by the Community Land Model. *Biogeosci. Discuss.* **7**, 565–630 (2010).
